# Exploring Parent and Autistic Youth Perspectives to Inform Adaptations for an Advocacy Program

**DOI:** 10.1007/s10882-025-10005-z

**Published:** 2025-01-29

**Authors:** Meghan M. Burke, Amanda N. Johnston, Saury Ramos-Torres, Sanskriti Singh, Leann Smith DaWalt, Julie Lounds Taylor

**Affiliations:** 1 Department of Special Education, Vanderbilt University, Nashville, TN USA; 2 Waisman Center, University of Wisconsin at Madison, Madison, WI USA; 3 Department of Pediatrics, Vanderbilt University Medical Center, Nashville, TN USA; 4 Department of Special Education, University of Texas at Austin, 2515 Speedway, Austin, TX 78712 USA

**Keywords:** Autism, Services, Advocacy, Navigation

## Abstract

Autistic Youth and their parents often struggle to access adult disability services. It is important to garner feedback from autistic youth and their parents to inform interventions to facilitate access to adult services. In this study, 128 parents of transition-aged autistic youth and 78 autistic youth completed individual interviews to inform adaptations to an advocacy program designed to improve access to adult services. Parents reported wanting a navigator (versus a group) program to help them learn to navigate adult services. However, parents reported concerns about the quality of navigators. Autistic youth reported wanting to learn about diverse issues in adulthood including types of adult services; most youth wanted to learn about services via videos. Implications for research and practice are discussed.

Across the lifespan, parents report struggling to access services for their autistic son or daughter. Ranging from services in early intervention (Schraml-Block & Ostrosky, 2022), school (Pearson & Meadan, 2021), and adulthood (Shattuck et al., 2020), parents often struggle to identify, apply for, and access services. Adult services may include: Social Security Disability Insurance (SSDI), technology, employment support, legal protections, special needs trusts, Medicaid waiver services, and housing vouchers (Taylor et al., 2017). Adult services may be especially difficult to ascertain. Unlike early intervention and school services which are often housed in Departments of Human Services and/or school systems, adult services are located in silos with varied eligibility requirements, norms, and bureaucracies. Parents of autistic individuals especially report that adult services are inaccessible, difficult to obtain, and often inadequate (Marsack-Topolewski & Weisz, 2020).

Because of the difficulty in accessing services, there is a need for interventions to support parents and autistic individuals in navigating service delivery systems. For parents of autistic children, some interventions have begun to surface that facilitate service navigation. For example, FIRME (Families Included in Receiving Better Special Education Services or Familias Incluidas en Recibiendo Mejor Educación Especial) is a 12-hour advocacy program educating and empowering Latino parents of children with autism about school services (Rios et al., 2021). In FIRME, parents learn in a cohort format about school services and advocacy skills. In a randomized controlled trial, FIRME was demonstrated to effectively increase special education knowledge, advocacy skills, and empowerment (Rios et al., 2024). In comparison, the Volunteer Advocacy Project (VAP) educates individuals, primarily parents of children with disabilities, to advocate not only for their own children but also for other parents to access special education services (Burke et al., 2019a, b). Via a 36-hour advocacy program about school services and non-adversarial advocacy skills, VAP participants learn together in a cohort to help other parents access services. In a randomized controlled trial of the VAP, participants demonstrated significantly improved special education knowledge and self-mastery (Burke et al., 2019a, b). VAP participants have gone on to support parents of children with disabilities in accessing educational services and improving family quality of life. In one of the few interventions which aims to improve access to adult disability services, Aleman-Tovar and colleagues (in press) tested ASISTIR (Apoyando a nueStros hIjo/as con autiSmo obTener servIcios de tRansición; Supporting our Children with Autism to Obtain Transition Services), a 12-hour program to increase knowledge, empowerment, and advocacy among Latino parents; results of a single-group study indicate that participants demonstrate significantly improved knowledge of school and adult disability services, advocacy, and empowerment.

Unlike FIRME and the VAP, some interventions for parents of autistic children utilize individual (versus group) formats to facilitate access to services. In navigator programs, a parent has a navigator who works with that parent individually to ensure their access to services. To date, navigator programs have yielded positive results demonstrating that access to a navigator can improve access to services for newly diagnosed autistic children (Feinberg et al., 2016) and school-aged children with autism (Magaña et al., 2020). One such navigator program is Promotoras en Acción (Parents Taking Action, PTA), which connects navigators (i.e., Latina mothers of children with autism) with Latina parents of young children with autism (Magaña et al., 2020). Research demonstrates that the promotoras (i.e., navigators) empower and educate parents, enabling them to access needed services (Magaña et al., 2020). However, there are several gaps in the extant research. For example, most programs focus on parents of autistic children—not parents of autistic youth or adults. Because adult services are located in silos, autistic youth and adults often rely on their families for service navigation (Taylor et al., 2017). To our knowledge, no research has explored whether parents prefer a certain type of program (i.e., group training or navigator) to learn about adult services. There may be tradeoffs between group training or navigator interventions with the former offering peer support and using cohort models and the latter being more individualized and intimate. By understanding the ways in which parents want to learn about adult services, interventions can be aligned to meet their needs.

Relatedly, it is important to explore the preferences of autistic individuals about how they want to learn about adult services. To date, most programs about adult disability services have focused on educating parents—not youth with disabilities (Author, submitted). Yet, the youth are the recipients of the services. Further, autistic adults report struggling to access services given systemic barriers (e.g., fragmented services, incompetent care, Maddox et al., 2020). Autistic adults report that, despite needing services, they often do not receive support (Micai et al., 2022). In alignment with the constructs of self-determination and self-advocacy (Shogren et al., 2017), youth with disabilities should take the reins of their own advocacy in the adult service delivery system. A beginning step to developing an intervention that educates autistic youth about adult services is to ask their perspectives about relevant content to be covered in an intervention.

Given the need for services in adulthood, it is crucial to create interventions for autistic adults and parents to navigate services. Although many youth struggle during the transition to adulthood, autistic youth are at greater risk across multiple domains of daily life. For example, autistic youth are likely to be unemployed or underemployed, living in segregated settings and/or lacking meaningful community engagement (Shattuck et al., 2012). By garnering feedback from autistic adults and their parents, interventions can be developed to meet their needs.

In this project, autistic adults and their parents were asked about their preferences to learn about adult service delivery systems. Specifically, participants were asked whether they would prefer a group-based program or a navigator to help access adult services. Parents were asked about the training a navigator would need to be effective in supporting other parents. Autistic youth were asked about how they wanted to learn about adult services. Notably, all parent participants completed ASSIST (Advocating for Supports to Improve Service Transitions). ASSIST is a 24-hour, 12 week program for parents of transition-aged youth with autism to learn about adult services and develop advocacy skills. ASSIST is conducted in cohorts. At the end of the program, parent participants and their youth were interviewed about their perceptions of ASSIST (for parents) and how they would like to receive information to access adult disability services (for parents and youth). Our research questions were: When deciding between a group-based program (i.e., ASSIST) and a navigator, (a) How do parents want to learn about adult services?; (b) According to parents, what training would navigators need to support families to improve access to adult services; and (c) How do autistic youth want to learn about adult services?.

## Method

### Participants

The participants in this study were a subset of participants in the larger multi-site randomized controlled trial of the ASSIST program. To be included in the larger study, parents needed to meet the following inclusionary criteria: identify as a parent or legal guardian of an autistic youth (aged 16–26), provide documentation of an autism diagnosis from a professional, be able to complete research measures in English and participate in ASSIST which is offered in English, have lifetime scores on the Social Communication Questionnaire (Rutter et al., 2003) of 12 or greater indicating a likelihood of autism, live in IL, TN or WI (the states wherein the study was conducted), and be willing to attend a 12-week advocacy program about adult services.

For this study, additional inclusionary criteria required the parent participants to have completed the ASSIST program. For the autistic youth, an additional inclusionary criterion was to have some form of communication (e.g., spoken language, sign language, an augmentative and alternative communication device). Altogether, for this study, participants had to complete the final interview about their experiences in the project.

In this study, there were 128 parents of transition-aged youth and 78 autistic youth (*N* = 206) who were part of the initial randomized controlled trial and completed the data collection for the research questions. The participants reflected three states (WI, TN, IL). On average, at baseline (i.e., before participants were randomized), parents were 51 years old (*SD* = 6.44, range 36.2–70.6). Also at baseline, autistic youth were, on average, 19 years of age (*SD* = 2.67, range 15.80–26.60). Many parent participants were White (*n* = 102) and identified as mothers (*n* = 118). Youth participants were mostly male (*n* = 55). See Table 1.

**Table 1:** Participant demographics

	% (*n*)
Parent Characteristics	
Gender: Female	89.84% (115)
Marital status: Married	67.97% (87)
Educational background	
Received high school degree	4.69% (6)
Some college	15.63% (20)
Associate’s degree	9.38% (12)
Bachelor’s degree	36.72% (47)
Master’s degree	28.91% (37)
PhD or profesional degree	4.69% (6)
Race or ethnicity	
White	78.13% (100)
Black or African American	10.94% (14)
Asian	4.69% (6)
Other	6.25% (8)
Hispanic or Latino: Yes	9.38% (12)
Annual Household Income	
Less than $40,000	9.38% (12)
$40,001–60,000	15.63% (20)
$60,001–80,000	12.5% (16)
$80,001–100,000	11.72% (15)
$100,001–125,000	10.94% (14)
$125,001–150,000	9.38% (12)
$150,001–200,000	12.5% (16)
More than $200,001	10.16% (13)
Youth Characteristics of the Youth who Participated in this Study	
Gender: Male	71.41% (55)
Educational status: Out of high school	34.61% (27)
Current Residence: In family home	93.59% (73)
Race or ethnicity	
White	78.21% (61)
Black or African American	12.82% (10)
Asian	8.97% (7)
American Indian	3.85% (3)
Other	6.41% (5)
Hispanic or Latino: Yes	10.26% (8)

### Recruitment

For the overarching project, several methods were used to recruit participants. For example, participants learned about the project from school personnel, research registries, disability agencies, and autism support groups. Information about the study was distributed via email, word of mouth, and recruitment flyers. Participants were compensated for participating in data collection.

### Procedures

Interested individuals could contact the research team or complete an electronic form to indicate interest. Telephone/video conferencing screening was used to determine whether potential participants met the inclusionary criteria for the study. If the individual met the inclusionary criteria, then they completed a baseline survey and interview (either in-person or via Zoom). After completing the baseline survey and interview, participants were randomized to either the treatment or waitlist-control condition (1:1 ratio) using a computer-generated list of random numbers generated by the biostatistician.

The treatment group received the ASSIST program. Due to COVID-19, the ASSIST program was delivered in a group format via Zoom. The parents in the waitlist-control group received all of the ASSIST curricular materials. After a 12-month waiting period, the parents in the waitlist-control group had the opportunity to take the ASSIST program virtually. A 12-month waiting period was to provide enough time to see an effect on service access. Notably, only parents participated in the ASSIST program; youth did not participate directly in the program. However, parents were encouraged to share ASSIST content with their youth and complete a letter of intent with their youth. Thus, youth may have some familiarity with the program.

Throughout the study, participants (inclusive of parents and youth) completed data collection. For this study, we focused on the data collected at the end of the project period which occurred approximately one year after the control group completed the ASSIST program and about 2.25 years after the treatment group completed ASSIST. Participants (parents and youth) completed individual interviews, respectively, garnering their feedback about the ASSIST program and potential adaptations.

### Description of the ASSIST Program

Complete information about ASSIST can be found in another publication. ASSIST is a 12-week, 24-hour program (i.e., two-hour sessions per week) that teaches parents about adult disability services. ASSIST is facilitated by an individual with knowledge about adult services. ASSIST topics included: person-centered planning, supplemental security income, SSDI, Medicaid waivers, employment, post-secondary education, legal decision-making, housing, special needs trusts, and advocacy. At the beginning of each session, all participants watched a 10–15 min video showcasing national information about the relevant topic. After watching the video, a state-specific expert from a local organization addressed the topic and its intricacies for the given state (i.e., IL, TN or WI). State-specific experts included representatives from The Arc, attorneys, and directors of community-based organizations.

### Measures

#### Baseline Survey

At baseline, all parent participants completed a survey about their backgrounds (e.g., race, ethnicity, socioeconomic status) to characterize the sample.

#### Interview Protocol for Parents

At the end of the project, the research team created an interview protocol for parent participants. For this study, the protocol questions reflected extant literature about advocacy for adult services (e.g., Taylor et al., 2017), navigator programs (e.g., Feinberg et al., 2016) and other perceptions of the ASSIST project. The research team reviewed the protocol multiple times. On average, parent interviews lasted 50 min (*SD* = 17.53, range 17–118 min). Sample questions included: “Compared to taking a class about adult services (like ASSIST), how helpful would it be to meet with a navigator who could work with you towards getting adult services for your youth? Why do you feel this way?”, “After taking ASSIST, how confident would you feel being a navigator for other parents?”, “What additional training would you need to be more confident being a navigator for other parents?”, and “If we have ASSIST navigators, would you be interested in being a potential navigator?”.

#### Interview Protocol for Autistic Youth 

The research team created a separate interview protocol for youth participants. The protocol was based on literature about self-advocacy (e.g., Shogren et al., 2021) and adult services (Taylor et al., 2017). Accommodations were made for autistic youth to participate in the interview (e.g., giving breaks, rephrasing questions, sharing the questions on the screen, using closed captioning). On average, youth interviews lasted 23 min (*SD* = 7.62, range 7–41). For example, the interview protocol started with open-ended questions such as “If we made a program for autistic individuals rather than parents what kind of program would be interesting to you?”. Then, the interviewer reviewed each type of service covered in the ASSIST program specifically asking youth whether they wanted to learn about that service. The interviewer stated: “Here is a list of different things we could talk about in this program. Please tell me which ones you would want to learn more about and why”. Topics included: different types of housing options and technology (i.e., housing and technology), governmental programs that can support living in the community (i.e., Medicaid waivers), and options for keeping money safe (i.e., special needs trusts and/or ABLE accounts). Youth were also asked how they would like to learn about services.

### Data Analysis

Interviews were transcribed verbatim. Three research team members individually read the transcripts numerous times to familiarize themselves with the data. Interviews were analyzed using emergent design (Patton, 2002). Specifically, we did not have an a priori coding system. Instead, we openly coded the responses. Using a line-by-line approach, the team individually coded all data. Each piece of data was compared to other data, highlighted, and given a code (Creswell, 2003). The team checked the existing codes to see whether the data belonged to an extant code or needed a new code. The team met to compare codes and resolve disagreements. Based on their analysis, the team created a codebook. Then, the team reviewed the data again using the codebook. After all data were coded, the codes were grouped into categories and organized into themes. Frequency counts were conducted with the themes to show their prevalence.

For example, the team coded youth responses about the type of information they wanted to learn about from a program about adult services. Initial codes included: employment, social activities, life skills, academic, and autism. At the initial coding stage, there were 56 agreements and 22 disagreements between the coders. Most disagreements related to coding for “budgeting” and “housing”; the coders discussed those codes and agreed that budgeting and housing should fall under “life skills”. For all disagreements, the coders discussed their differences and reached consensus. They finalized the codebook and returned to analyze the data. They grouped the codes into categories and themes.

### Trustworthiness

Several efforts were made to ensure the trustworthiness of data collection and analysis. For data collection, the interviewers were trained in the interview protocol and had experience working with autistic youth and parents. The interviewer was not involved in the delivery of the ASSIST program. Accordingly, the participant may have felt more comfortable providing honest and, potentially, negative feedback about the ASSIST program. For data analysis, the research team conducted peer debriefing via weekly team meetings to discuss the coding. Also, negative case analysis was conducted when analyzing the data (Brantlinger et al., 2005). For example, if a topic was suggested by a participant, we reviewed all transcripts to identify whether any data disconfirmed that suggested topic. Pseudonyms were used for all participant names.

### Researcher Reflexivity

The research team was comprised of three faculty members, a post-doctoral study coordinator, and two graduate students. All research team members have experience working with individuals with autism and their parents. Further, two of the research team members have family members with disabilities. The team included two individuals of color; all team members identified as female. None of the team members identified as having disabilities. Altogether, the team believed in the value of ASSIST for supporting autistic youth and their parents in accessing services. The team recognized that their bias may influence their perceptions. Accordingly, throughout data collection and analysis, the research team reflected together and via individual field notes about their biases.

## Findings

### Most Parents Reported Wanting Navigators for Adult Services

Altogether, 82.81% (*n* = 106 of the 128 participants in this study) of parent participants reported that a navigator would be “more helpful” than the ASSIST program, 13.28% (*n* = 17) reported that a navigator would be the “same or no difference” as the ASSIST program, and 3.90% (*n* = 5) reported that a navigator would be “less helpful” than the ASSIST program. Some participants freely reported whether they would want a combination of the ASSIST program and a navigator. Across the parent participants, 42.19% (*n* = 54) reported that they would prefer a combination of a navigator and the ASSIST program, 29.69% (*n* = 38) reported they would only want a navigator, and 28.13% (*n* = 36) reported they were unsure about having a combination of a navigator and the program.

#### A Navigator would be more Helpful than ASSIST

Of the 106 participants who reported that a navigator would be “more helpful” than the ASSIST program, many participants reported that navigators could provide individualized support to parents. Describing the potential benefit of having a local navigator, Katie, a white mother with a graduate degree living in Illinois, reported, “Each parent has different levels of knowledge and understanding of the system. Navigators can target the needs of different parents more personally to understand and fit the unique needs of each parent. Navigators will be someone locally that parents can go back or call if parents have any questions.” Similarly, LaTonya, a Black mother in Tennessee who attended some college, reported, “[Having a navigator would be] more helpful since it would be more like an IEP that is there just for your child - not everything in [the ASSIST] class was applicable”. Additionally, some participants reported that navigators could tailor their assistance depending on the logistical needs of parents. Janet, a mother from Wisconsin, stated, “If there were navigators available, that would be very helpful… If you’re working a full-time job and trying to navigate services, it is really difficult. Trying to make all of those pieces fit during a work day is really challenging. A navigator would really helpful”.

Some parent participants reported that the preference for attending the ASSIST program versus a navigator depends on the unique needs of the family. Amy, a white mother with a master’s degree living in Tennessee, reported, “[A navigator] would be really great… [but] giving them [parents] the option since some people like to do things themselves or maybe they have multiple kids and are busy; whatever is best serving their situation”. Similarly, Lilly, a mother from Wisconsin, reported preferring a navigator to the ASSIST program. However, Lilly reported that, for other families, the choice between the ASSIST program and a navigator would depend on the families’ circumstances: “[A navigator could put] less demand on the parents’ time. Depending on where their child falls on the spectrum, it will narrow down what [information] applies to which [family, and families could] avoid learning about the wealth of info they don’t really need”.

#### A Navigator would be the same as ASSIST

Of the 17 participants who reported that having a navigator would be “the same or no difference”, most participants reported that they would prefer a combination of having a navigator and attending the ASSIST program. Natalie, a white grandmother with some college education from Tennessee, reported, “You need both [ASSIST and a navigator]. [You] cannot do one without the other. You cannot use [a] navigator to read their mind. People need basic knowledge [such as the ASSIST program]”. Other participants similarly reported that navigators would be beneficial if they were combined with the ASSIST program. Jenny, a white, married mom with a high school degree from Wisconsin, reported that the combination of the ASSIST program and a navigator could be helpful. In response to what would be most helpful, Jenny reported, “A combination of both during the transition out of high school. It was great to have the knowledge, but also someone to reach out to with specific questions [would have been helpful]”. Reflecting on the importance of having knowledge about adult services and someone to guide them through the service delivery system, Pearl, a Black mother who worked as a social worker in Illinois, reported “A navigator would have the information and the parent wouldn’t; thus inhibiting the parent’s ability to gain knowledge for themselves. Some parents would like to have a navigator to do the work for them. Families would need the ASSIST program first then a navigator.”

Similar to the participants who preferred a navigator over the ASSIST program, some participants who reported “no difference” between the ASSIST program and a navigator also suggested that the preference for a navigator or the ASSIST program depended on family circumstances. Paula, a white mother with a master’s degree from Tennessee, reported “We did not really need that [navigator], but I can see how that would be extremely useful for people who are overwhelmed with [the] care of loved ones”. Indeed, many participants explained that the need for a navigator (versus the ASSIST program) depends on what works best for families, especially in terms of accessing information about adult services. Carina, a divorced mother with an associate’s degree who worked as an advocate and lived in Wisconsin, reported “[Having a navigator] would have been a good contrast, but I appreciated the program as it was structured. ASSIST worked because I could do it from home–it was accessible. Having it available online maintains accessibility. It is easier for parents to stay at home, so a Navigator would be great if you can accommodate this”. Participants reported that either the ASSIST program or a navigator allowed parents to network with many other people. Sarah reported that both options would allow parents to be “plugged in [to information] and have a good support group and resource center with colleagues/friends”. In responding to whether a navigator would be helpful, Allie, a White mother with a college degree who worked as an aide from Wisconsin, stated “It depends on your personality. I’m shy so I loved having it online. I loved that I could be anonymous and soak in the information. I’d be uncomfortable having someone come into my home.”

#### A Navigator would be less Helpful than ASSIST

Of the five participants who reported that a navigator would be “less helpful”, participants described that it depends on the quality of the navigator. Marnie was a white mother living in Wisconsin. She had a master’s degree. She reported, “It would depend on: who the navigator was, who is paying for the navigator, [and] the experience of the navigator”. Other participants also commented on the quality of the navigator. Put simply by Karla, a white mother who worked as a nurse: “they [the navigator] might not have time or interest [to support families]”. One participant reported that the ASSIST program would be more helpful than a navigator because ASSIST allows parents to network with one another. Anna, a white, college-educated mother living in Illinois, reported that having a navigator “may be more time-consuming [than the ASSIST program]”. She elaborated that she, “likes to talk to other moms and listen to their experiences”. One participant reported that a navigator may be overwhelming. Darla, a mother from Tennessee, described having a navigator as overwhelming: “A one-on-one [such as a navigator] would have been a bit much; attending classes was a better option”.

### Perceptions about being a Navigator

Overall, 80.47% *(n* = 103*)* of parent participants reported that they wanted to be navigators and 42.19% (*n* = 54*)* reported that they would feel confident being navigators. When asked, “What additional training would you need to be more confident being a navigator for other families?”, 38.28% (*n* = 49*)* reported that they would need more training. There was diversity in the types of training suggested by the participants. Topics included: more information about ASSIST topics *(*65.79%, *n* = 51), ways to match service needs to the family *(*45.15%, *n* = 35), and general information that would assist families (e.g., handbook of terms/services, local resource guide) (42.7%, *n* = 33). Less frequent topics included: learning about families’ experiences navigating services, (18.06%, *n* = 14), updated information about the application processes to access adult services (11.61%, *n* = 9), advocacy about governmental services (10.32%, *n* = 8), ways to answer questions about specific services (9.03%, *n* = 7), in-person networking with other families (9.03%, *n* = 7), information about special education services (2.58%, *n* = 2), and resources related to self-care and mental health (2.58%, *n* = 2). See Table 2 for examples of each topic.

**Table 2:** Suggested topics for a Navigator Training

Training Topic	Example Quote
ASSIST Topics	“I would probably need more training. Each [ASSIST] class had a lot of information, if it were to happen now, I would need to be “re-trained” with a different mindset and revisit the book. I only know what I know about myself in that process, so I would need to be re-trained.”.
Ways to match parents’ service needs to the parent	“One on one training - Written material more than videos and Comparison charts with agencies, systems, and who does what to lay out all the information.”
General information	“It would need to be set up for parent. Here’s the book, here’s how you do it. Need to be where parent would have all the answers what might come up in a set up.”
Other parents’ experiences navigating services	“Work with other families who have been in the program and review the materials together. Talking through different scenarios.”
Information about application processes	“Make sure she understood all the forms and would need to go through every application in depth (what are they looking for and how to fill things out.”
Advocacy for governmental programs	“More about the specific governmental pieces and understanding how services are being funded in order to help families know what they need to advocate for on a legislative and democratic level.”
Questions about specific services	“What DRS does. Information about that Medicaid wavier”
Networking with other parents	“Expect additional training and virtual groups and learning in real life and communicating, visiting offices and real-life connections, learning enough to be a navigator -has lost touch with special needs/disability community and would like to get involved again to see if its a good fit.”
Special Education information	“IEP and 504; most families need this information.”
Self-care and mental health	“All trainings need to incorporate self-care; it is really easy to get exploited in this area, so you need to be in a good place and have boundaries about what you are and are not going to do; there is so much need and easy to sacrifice yourself.”

### Youth Perceptions of ways to Learn About Adult Services

When open-endedly asked the content of an advocacy program for autistic youth (versus parents), autistic youth reported diverse responses. The most frequently reported topics included: employment (*n* = 14, 17.95%), social activities (*n* = 10, 12.82%), and life skills (*n* = 9, 11.54%). The less frequent topics were: academic content (*n* = 5, 6.41%), and autism (*n* = 5, 6.41%). However, several participants reported more vague responses including: unsure (*n* = 15, 19.23%), nothing specific (*n* = 9, 11.54%), no answer (*n* = 6, 7.69%), other (*n* = 3, 3.85%) and answers not related to the question (*n* = 2, 2.56%). See Table 3 for examples of each of the responses.

**Table 3:** Topics that autistic youth reported Wanting to learn about

Topic	Example
Employment	-“Mostly a program that could teach during job how to handle sensitivities and over stimulation; how to cope with it acceptably.” -“Job.”
Social activities	-“Relationships”. “Do more programming on dating and relationships.” -“An outing- like day at the movies.”
Life skills	-“Think my biggest problem is getting accommodations for places…like if first contact does not respond or is not helpful or does not answer…who to go from there”. -“Maybe one where you learn about budgeting or even learning how to cook on your own since he has never really had to; he can make breakfast for himself like toast and eggs.”
Academic	-“It could be like train engineering.” -“Reading program”.
Autism	-“Program for autistic adults, not really for youth anymore.” -“Knowledge about different types of autism”.

After responding to the open-ended question about the content of an advocacy program, youth were specifically asked whether ASSIST topics would be relevant to them. Participants reported that they would like to learn about the following ASSIST topics: employment or post-secondary supports (*n* = 59, 75.64%), keeping money safe (*n* = 57, 73.08%), governmental programs for financial assistance (*n* = 55, 70.53%), governmental programs for daily living (*n* = 50, 64.10%), person-centered planning (*n* = 50, 64.10%), legal protections about decision-making (*n* = 47, 60.26%), housing (*n* = 47, 60.26%), legal protections for health decision-making (*n* = 45, 57.69%), health insurance (*n* = 42, 53.85%), and technology (*n* = 41, 52.56%).

When asked why each youth wanted to learn about specific services and supports, there were some consistent and some varied answers. Across all services and supports, some youth reported that learning about that given service or support would be “helpful”, “important”, “useful” and/or “interesting”. Another relatively consistent response was needing to learn about certain services and supports due to their own lack of knowledge. For example, 22.81% (*n* = 13) reported needing help with money management and thus wanting to learn about keeping money safe. Further, 21.81% (*n* = 12) of the youth participants wanted to learn about governmental programs for financial assistance because the youth reported “needing” financial assistance. With respect to housing, 48.94% (*n* = 23) of youth participants reported needing housing in the future. Similar responses about “needing” to learn about services and supports occurred for: legal protections for decision-making in healthcare, health insurance, and technology.

There were also varied answers relevant to specific domains. For example, of the 59 participants who said they wanted to learn about employment or post-secondary supports, 64.41% (*n* = 38) reported that learning about these supports would enable them to have employment and/or attend their desired post-secondary program. With respect to keeping their money safe, 26.32% (*n* = 15) reported wanting to learn about keeping their money safe so no one could steal their money. Regarding governmental programs, 44% (*n* = 22) reported wanting to learn about governmental programs so they could do certain things in the future (e.g., cook for themselves, navigate transportation). In reference to person-centered planning, 40% (*n* = 20) wanted to learn about person-centered planning so they could be better self-advocates. Interestingly, 14.89% (*n* = 7) wanted to learn about housing as they were interested in moving to a different state in the future. See Table 4.

**Table 4:** Types of ASSIST services Autistic Youth wanted to learn about

Service and percentage who wanted to learn it	Reason for wanting to learn about it*	Sample quote	Reasons for not wanting to learn about it	Sample quote
Employment or post-secondary supports (*n* = 59, 75.64%)	-It would help me get the job that I want and/or attend the post-secondary program I want (*n* = 38).	“This would help me find a job that is attuned to my interests where I would be comfortable”.	-I do not need it (*n* = 8).	-“ I am pretty good on my own. I am surprisingly good at networking”.
	-I want to learn about it because it would be useful (*n* = 4).	-“I’d say its something that would be very useful”.		
Keeping money safe (*n* = 57, 73.08%)	-I would learn so people do not steal my money (*n* = 15).	-“People can steal and I want to keep my things safe”.	-I know how to manage money (*n* = 5).	-“I have that down already”.
	-I need help with money management (*n* = 13).	-“ I don’t know how good I will be with managing money”.	-My family helps me manage money (*n* = 3).	-“I have been talking to my dad about how to pay bills”.
	-This would be a useful topic (*n* = 9).	-“That would be of high interest”.		
Governmental programs for financial assistance (*n* = 55, 70.53%)	-It would be helpful to know about financial assistance (*n* = 23)	-“Sounds useful”	-I do not need it (*n* = 5)	-“I hope I will have a good job with the physics gig”
	-I need financial assistance (*n* = 12)	“I am broke. Finding a job is hard.”	-I don’t feel like sharing my reason (*n* = 1)	“I know why but I don’t feel like sharing”.
	-No reason (*n* = 3)	-“No reason”		
	-Managing money can be stressful (*n* = 1)	“Managing money on your own can be stress inducing”.		
Governmental programs for other supports in daily living (*n* = 50, 64.10%)	-It would help me do things I want to do in the future (e.g., cooking, transportation) (*n* = 22)	-“That would be good. Cooking classes especially are something I would like to do in the future”.	-I do not need it (*n* = 5)	-“I can do this on my own”.
	-It sounds useful for me to be independent (*n* = 8)	-“The more independent, the better”.	-I would rather learn on my own (*n* = 1)	-“I can get the feel myself so I would rather learn on my own”.
	-I would like to learn about it (*n* = 7)	-“I would like to learn about that”.	-I don’t feel like sharing why not (*n* = 1).	-“I know why but I don’t feel like sharing”.
Person-centered planning (*n* = 50, 64.10%)	-I want to learn more about person-centered planning so I can be a better self-advocate (*n* = 20).	-“Yes, self-advocacy skills”.	-I do not need help with person-centered planning (*n* = 5).	-“I am already good at this”.
	-The topic sounds useful and interesting (*n* = 10).	-“Sounds useful”.		
Legal protections about decision-making (*n* = 47, 60.26%)	-I want to learn more about this topic because laws are tricky (*n* = 10)	“The laws where they are set up are a huge mess and it’s important to learn how to navigate them because it’s impossible to do without help.”	-I do not need it as I am independent (*n* = 2)	-“Not for me, I can make my own decisions”
	-I want to learn more about this is an important topic (*n* = 3)	“[I just] think it is important”.	-Dislike learning about the law (*n* = 2)	-“I don’t like legal stuff.”
	-Because my parents help me with my decisions (*n* = 3)	-“So I do not always have the pressure to do it on my own”.	-I do not need it because my parents have already addressed it (*n* = 1)	-“My parents have taken care of it”.
	-I think this information will be helpful for my future (*n* = 3)	“So I can move on and get ready for my future”.	-Unsure (*n* = 2)	-“[I] don’t know why not”.
	-For safety (*n* = 1)	--“So I am safe”		
	-Unsure (2)	-“I don’t know why”.		
Housing (*n* = 47, 60.26%)	-I will need housing in the future (*n* = 23).	-“ I have been putting a lot of thought into that like getting a house after my PhD”.	-I do not need it because I like living at home (*n* = 5).	-“Not useful to me personally because I like living at home”.
	-I would like to learn about it because it is interesting and/or important (*n* = 7).	-“I would like to learn about it but I like living at home”.	-I do not think it will be hard for me to figure out housing on my own (*n* = 2).	“I don’t think its complex. Just deciphering it [housing] and getting things set up.”
	-I would like to learn about housing as I want to move to another state (*n* = 7).	-“I’d like to move to Florida where it is always sunny”.		
Legal protections for health decisions (*n* = 45, 57.69%)	-It is important (*n* = 4)	-“It’s important”.	-I do not need it because I can do it on my own (*n* = 2)	-“I am pretty comfortable already”.
	-Due to past experience, this is needed (*n* = 4)	-“I had a doctor discriminate against me because I was asking to get my tubes tied and doctor said ‘I needed to be in right mind’. Ableism is real”.	-This is not an interesting topic (*n* = 1)	-“Not so interesting”.
	-I may need help with doctors (*n* = 2)	“If I end up in the position where I am not sure if I need to go to the doctor, I can ask my family for an appointment”.	-I do not need it because my family already helps me (*n* = 1)	-“I leave it to my parents”.
Health insurance (*n* = 42, 53.85	-I need to learn about health insurance (*n* = 13)	“I am still on dad’s [insurance] so I need to learn about this.”	-I do not need help because my family does this for me (*n* = 3)	-“My mom is a nurse and was involved in helping me with the application process”.
	-I am interested in this topic (*n* = 6)	-“Good topic”.	-I do not need this as I already have health insurance (*n* = 2)	-“I already have Medicaid”.
	-I want to learn more about healthcare (*n* = 5)	-“The more I can learn about health care [the better]”.	-I am not interested in learning about health insurance (*n* = 2)	-“I am not interested in it”.
	-I want to pick the right health insurance (*n* = 2)	“I want to pick the right health insurance because they are not all the same”.	-Health insurance is expensive (*n* = 1)	-“Health insurance is very expensive”.
Technology (*n* = 41, 52.56%)	-I need and/or like technology (*n* = 13).	-“I am better with iPads than anything else”.	-I am already good with technology so I do not need more help with it (*n* = 14).	-“Pretty good with that [technology]”.
	-This topic interests me (*n* = 9).	-“Very useful topic”.	-I am uncomfortable with technology (*n* = 1).	-“I am weary of 3rd party apps and technology”.
	-I use technology so I want to learn more about technology (*n* = 7).	-“I use a speech generator so I am more interested in this topic [vs. to others]”.		

*Not all participants provided a reason for why they did or did not want to learn about a given topic

Youth also responded about their reasons why they did not want to learn about certain services and supports. Across each service and support, some youth participants reported “not needing” to learn about a service and support. Further, with respect to keeping money safe, legal protections about decision-making, legal protections for decision-making in healthcare, and health insurance, some youth participants reported not needing to learn about the service or support because their parents had already taken care of the service or support. There were also some varied responses across different content areas. With respect to housing, 16.13% (*n* = 5) of youth participants reported not needing to learn about housing because they liked living in their family home. Unique to technology, 37.84% (*n* = 14) of youth participants reported not wanting to learn about technology because they were already adept at using technology.

When asked about the way in which youth would want to learn about services and supports, most youth wanted to learn by watching videos. Specifically, 39.74% (*n* = 31) of youth wanted to watch videos. To a lesser extent, other mediums were also suggested such as: having an autistic adult talk with you one-on-one (*n* = 13, 16.67%), getting together with a group of peers to talk (*n* = 10, 12.82%), participating in interactive activities (*n* = 8, 10.26%), reading informational materials (*n* = 7, 8.97%), and listening to a speaker in-person (*n* = 7, 8.97%). Two participants reported no preference.

## Discussion

It can be difficult for autistic youth and their parents to access adult services. To improve service access, it is important to glean input from autistic youth and parents to develop tailored and responsive interventions offered in their preferred format with desired content. In this study, we explored the perspectives of autistic youth and parents who participated in ASSIST, a group advocacy program. We had three main findings. First, most parents reported that they preferred a navigator more than a group advocacy program. While group advocacy programs can significantly improve access to services (e.g., Burke et al., 2019a, b), many participants reported that they preferred having a navigator. Notably, such responses occurred among parent participants who all had the option to participate in a group advocacy program. While not applied in the context of adult services, research has shown that navigators can improve access to services among parents of autistic children (e.g., Feinberg et al., 2016).

While most participants reported preferring a navigator (and many parents wanted a combination of the advocacy program and a navigator), many participants reported that family circumstances may dictate whether a navigator or group advocacy program may be appropriate. This finding suggests that a “one size fits all” approach to interventions to improve service access may be inappropriate; the choice between intervention types may depend on individual family needs. Notably, the suggested family circumstances that may impact such a choice varied ranging from parent personality type (e.g., shy parents may prefer a group advocacy program wherein they could be anonymous), a desire for a less intensive option, and/or accessibility (e.g., the preference to participate in a group advocacy program online from one’s home versus a navigator that could entail in-person contact). This finding suggests that it may be important to target certain types of intervention modalities to those most likely to benefit from them.

Second, while many participants were interested in becoming navigators, they also reported that they would need more training. Core navigator activities have been established (Broder-Finger et al., 2019). However, navigator training is less clear. Extant navigator programs report having different options for navigator training ranging from professional training as a social worker (e.g., Feinberg et al., 2016) to a researcher-provided training for family navigators (Lopez et al., 2019). From this study, it seems that a 24-hour training about adult services would be insufficient to train navigators to support families.

Relatedly, if a navigator is provided, it is important also to provide a parent with peer support. Many participants reported wanting to receive peer support from other parents of autistic individuals. To retain peer support in a navigator model, the navigator could intentionally connect parents to autism support groups. Alternatively, the navigator could also be the parent of an autistic individual. To date, there are models wherein the navigator is also the parent of a child with a disability (e.g., Lopez et al., 2019). However, to our knowledge, no research has directly compared whether the lived experience of a navigator impacts their effectiveness. In spite of the lack of research, other studies suggest the importance of peer support (e.g., Solomon, 2004). More research is needed to identify ways for parents to still access peer support if they work with a navigator.

Third, there were diverse suggestions for the content of an advocacy program among autistic youth. Given the diversity of suggestions, it may be that youth would be interested in an advocacy program, like ASSIST, that covers a range of topics. Because of the heterogeneity in autism (Lord, 2019), sharing information about myriad topics may help ensure that an advocacy intervention can be responsive to the unique needs of each individual. Regardless of the types of services youth reported wanting to learn about, it is difficult to navigate any adult service delivery system (Carlson & Wilt, 2020). Research is needed to understand how to present complicated and nuanced information about services in accessible ways to autistic individuals.

### Limitations

While a jumping off point, this study had several limitations. For example, the study was based on cross-sectional data. It could be that parent and youth perceptions change over time; thus, a snapshot of their perceptions at one point in time may not be generalizable. Additionally, the participants were parents of transition-aged youth; findings may be different for youth who are younger than 16 or older than 26 years of age. Further, the youth participants did not take ASSIST. Without partaking in the group advocacy program, it may have been more difficult for them to answer questions about ways to learn adult services. Also, this study had limited transferability of the findings. Specifically, all parent participants had the option to complete the ASSIST program; thus, the findings may not be transferable to autistic youth and their parents who did not have the option of attending an advocacy program. Given that all parent participants had the option to take the ASSIST program, they may have been more knowledgeable and/or empowered than the general population. Further, the sample was not racially, ethnically or socioeconomically diverse; more research is needed to determine parent and autistic youth input from marginalized populations to ensure interventions are culturally responsive. Finally, participants did not have the opportunity to member check their responses; there may be greater credibility of the research if the participants could have engaged in member checking.

### Directions for Future Research

Future research may include an intervention study directly comparing the feasibility and effectiveness of a group advocacy program to an individual navigator. In doing so, such a study may shed light on the moderators of effectiveness (i.e., for whom is which intervention most feasible and effective). In the context of such a study, it is also critical to consider the potential for scalability and adoption by community-based organizations (Morrison et al., 2009). It may be cost and resource prohibitive to provide a navigator to every parent of a transition-aged youth with autism; however, by identifying for whom a navigator is most effective and feasible, community-based organizations can target specific autistic youth and parents who need individualized support.

Research is also needed to develop and test interventions so autistic youth can navigate adult services. Across all of the ASSIST-related topics, most youth participants reported wanting more information. Unlike ASSIST or a navigator program, youth participants overwhelmingly reported wanting to receive information via videos. The desire to receive information digitally (especially via videos) is common among transition-aged youth in the general population (Coyne et al., 2016). To this end, researchers may consider developing and testing a digital intervention that helps educate and empower autistic youth to access services.

### Implications for Practice

From this study, there are several implications for practice. At the most basic level, the findings suggest that community-based organizations may want to provide some supports and resources to autistic youth and parents to access services. All participants reported wanting some type of intervention to improve their access to interventions. Community-based organizations may especially consider the request for navigators. While providing navigators may be difficult, organizations may inquire within their own constituencies of who may want to serve as navigators. As demonstrated by this study, there may be several parents interested in serving as navigators; the next step will be for community-based organizations to offer training for individuals to become navigators.

## Data Availability

The data are available in the National Database for Autism Research (NDAR).
